# The Gold Standard Program for Smoking Cessation is Effective for Participants Over 60 Years of Age

**DOI:** 10.3390/ijerph120302574

**Published:** 2015-02-27

**Authors:** Mette Kehlet, Torben V. Schroeder, Hanne Tønnesen

**Affiliations:** 1 Vascular Clinic, RK, Rigshospitalet, Blegdamsvej 9, 2100 Copenhagen, Denmark; 2 Faculty of Health and Medical Sciences, University of Copenhagen, Blegdamsvej 3B, 2200 Copenhagen N, Denmark; 3 Centre for Clinical Education, Region Hovedstaden, 2100 Copenhagen, Denmark; E-Mail: torben.schroeder@regionh.dk; 4 WHO Collaborating Centres, Clinical Health Promotion Centre, Bispebjerg and Frederiksberg Hospitals, University of Copenhagen, Denmark; E-Mail: hanne.tonnesen@regionh.dk; 5 Clinical Health Promotion Centre, Lund University, Skåne University Hospital, Malmö SE 205 02, Sweden

**Keywords:** smoking cessation intervention, elderly, intensive program, Gold Standard Program (GSP), continuous abstinence, prospective cohort study, national database, Denmark

## Abstract

*Background:* Tobacco smoking is more prevalent among the elderly than among the young, and the elderly also have the most frequent contact with the health care system. The aim of this study was to evaluate the effectiveness of the Gold Standard Program, which is an intensive six-week smoking cessation program, on continuous self-reported abstinence rates after six months, on participants over the age of 60 years in a real life setting. *Methods:* This was a retrospective cohort study from the national Danish smoking cessation database. *Results:* The database registered 7369 participants over the age of 60 years (range 60–82) and 24,294 below 60 years (range 15–59). Continuous abstinence rate after six months was 37% for the elderly compared to 35% for the younger (*p* < 0.05). The significant variables for continuous abstinence were: living with another adult (OR 1.10), prior professional recommendation for smoking cessation (OR 1.12), being compliant with program (OR 1.35) and being abstinent at end of course (OR 13.3). *Conclusions:* Participants over the age of 60 years had significantly higher continuous abstinence rates after six months than the participants less than 60 years. It is never too late for health professionals to recommend and educate patients about smoking cessation programs even if they are over 60 years of age.

## 1. Introduction

Tobacco smoking is the biggest cause of illness and pre-mature death in the Western part of the world [1]. Worldwide it is estimated that more than five million people die from tobacco-related disease each year [2]. In Denmark the amount of daily smokers have diminished from 30% in 2000 to 17% in 2013 and approximately 1%–2% of Danish smokers successfully quit smoking each year [3]. Unfortunately there has not been an equal reduction in the amount of tobacco sold. A possible explanation for this could be that it was mainly the smokers who had the least tobacco consumption who succeeded in quitting smoking. Also, the reduction of smokers in Denmark has been more modest when compared to the other Nordic countries. Considering the age distribution, smoking seems most prevalent among 50–59 year-old Danes with 26% being daily smokers, whereas among citizens 60 years or older 16% are daily smokers [3].

It is a clinical experience that health professionals are less persuasive in recommending smoking cessation for patients over the age of 60, simply because they think this group of patients have smoked for too long and are not likely to quit at this age. But the people with the most frequent contact with the health care system are those over 60 years of age and therefore it is the group of smokers where there is the greatest potential for health care professionals to recommend, educate and influence them for smoking cessation programs. Thus, it is highly relevant to investigate the effect of smoking cessation intervention among this group.

The positive effects of smoking cessation have been shown for many years regarding almost all functions of the body from pulmonary function [4,5], reducing risk of cancer [6,7] and coronary disease [8,9,10], and intensive smoking cessation programs also have a positive effect on reducing the risks of having postoperative complications [11,12].

The aim of this study was to evaluate the effectiveness of intensive smoking cessation intervention programs (The Gold Standard Program, GSP) on continuous self-reported abstinence six-months after intended quit date on people from 60 years of age, and comparing this group of participants to the participants under the age of 60 years, as well as all participants in the Smoking Cessation Database. Furthermore we wanted to analyze selected variables gathered in the Smoking Cessation Database for a possible association with continuous abstinence.

## 2. Methods

### 2.1. Design

This is an observational cohort study using data from the Danish Smoking Cessation Database [13].

### 2.2. Setting

The Smoking Cessation Database is a database where more than 350 units in all 5 regions of Denmark contribute with data from smoking cessation programs. Both primary care facilities and hospitals are contributors to the database.

### 2.3. Material

Data have been collected in the Smoking Cessation Database since 2001. Since 2006 all entries has been identified with a unique 10-digit personal identification number (PIN) and therefore the database could be searched for doublets and for patients attending the program more than once. All participants gave written consent before entering the program.

**Figure 1:**
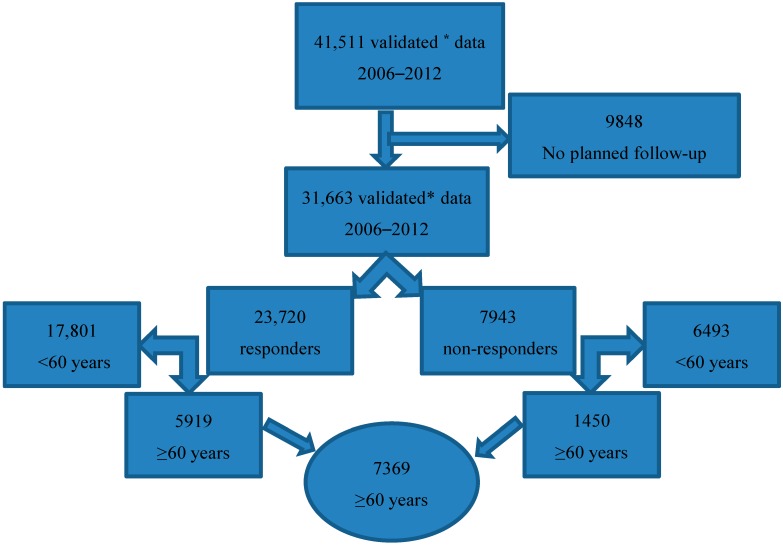
Flow-chart for the study population according to definition of age and responders *vs.* non-responders. * Validated data: doublets and non-existing personal identification number are excluded.

In this study we included participants attending the GSP in Denmark in the seven-years period January 2006 through December 2012, and where follow-up was intended. Analyses were conducted for participants in three pre-defined groups termed “elderly” (≥60 years), “young” (<60) and “all”. Before the analysis, the data was validated by searching for doublets and non-existing personal identification numbers and these were excluded from further analysis. After validating the data, the participants where follow-up was not intended were excluded (Figure 1).

The Smoking Cessation Database documents for each patient among others: The setting, the region in which the participant lives in, payment modality and whether the smoking cessation program is individual or group based. It also documents age, amount smoked per day, pack-years, Fagerström Test for Nicotine Dependence [14], previous attempts to quit, living with smoker *vs.* not living with smoker, compliant or non-compliant with program, way of recommendation, occupational position, level of education, and living status. For complete list of data collected, see Table 1.

The follow-up consisted of a telephone interview where 4 attempts were made to reach the patient where at least one call was during the evening. If the patient was not reached following this procedure the patient was reported as a non-responder.

### 2.4. Intervention

Since 2001 the Gold Standard Program (GSP) has been the smoking cessation program of choice in Denmark. It is an intensive smoking cessation program developed by the National Cancer Society and the National Stop Smoking Centre [15]. It was developed to uniform and optimize smoking cessation nationwide. It consists of a program with 5 meetings (individual or group-based) over 6 weeks with a pre-defined schedule of motivational conversation, education of the hazards of smoking, risks of relapse, thoughts and reflections on quitting, challenges during smoking cessation and ways to prevent relapse. All patients are scored according to the Fagerström test during the first meeting and are free to choose different kinds of nicotine replacement under the guidance of the smoking cessation instructor. The GSP is normally offered free of charge by the Danish municipalities and regions but most patients must themselves pay for nicotine replacement. To further enable their smoking cessation a telephone hotline is available so the patients can call in between meeting sessions.

**Table 1:** Data collected by the Danish Smoking Cessation Database.

Setting:	Hospitals and Pharmacies. County or Municipality.
Living Region:	Capital of Copenhagen, Zealand, Southern Denmark, Central Denmark, North Denmark.
Payment modality:	No free medication, free medication for a few days, free medication for <5 weeks or 5 weeks medication free of charge.
Smoking cessation program:	Individual or group based.
Age:	At entering program.
Amount smoked per day:	Cigarettes, pipes, cigars, others smoked per day.
Pack-years:	(Cigarettes per day × years of smoking)/20.
Fagerström Test for Nicotine Dependence 0–10 points:	0 point = No dependence. 1–2 point = Low dependence. 3–4 point = Low to moderate dependence. 5–6 point = Moderate dependence. 7–10 point = High dependence.
Quit attempts:	Number of prior attempts to quit.
Living status (socially):	Living with adult (>18 years), smoker, children.
Compliant or Non-Compliant with program:	Defined as attending >75% of meetings *vs.* <75%
Way of recommendation:	Self-referred or referred by health-professional.
Occupational position:	Working, disability, pensioner, retired, unemployed, student, other.
Level of education:	Primary school, high school, skilled craftsman, short, medium or longer higher education, other.
Living status (housing):	Home ownership, rental, other.
Smoking status at end of program:	Abstinent, continuous smoker, unknown status.

### 2.5. Outcome

The primary outcome was continuous self-reported abstinence 6 months after the date of intended quit date, or if one such did not exist, the last smoking cessation treatment date.

### 2.6. Statistical Analysis

Statistical analysis was done by using SPSS version 22. A univariate analysis was performed for the categorical and continuous variables listed in Table 2 and were assessed using the cross-table methods with Fisher’s exact test (two sided). This was done for all three pre-defined groups, “Elderly ≥ 60 years”, “Young < 60” and ‘All’. For the group of participants ≥ 60 years of age, the variables with a *p*-value < 0.1 were considered significant to be included in further analysis.

To identify the relative impact of the independent variables for continuous abstinence for the elderly, a logistic regression analysis was performed using the backward stepwise selection. In the multivariate analysis *p* < 0.05 was considered significant. The participants with “unknown” values (missing data) were excluded from the analyses. The numbers were considered small and acceptable for a real life study. All significant results are presented as odds ratios (OR) with 95% confidence intervals (CI).

All missing data and any loss of follow-ups were included, analyzed and reported according to the STROBE recommendations [16]. Data were also analyzed and reported according to the Russell Standards for RCTs [17], which assume that non-responders have relapsed and they are thereby included in the analysis as smokers, to ensure that this study can be compared to randomized studies. Therefore two columns for each of the three pre-defined groups are presented in Table 2; one column showing “All”, where non-responders are considered still smokers, and one column showing “Responders” only.

## 3. Results

In total 31,663 participants were entered in the database between the years 2006–2012. Of these, 7369 (23%) were 60 years or older when entering the GSP. Follow-up data was obtained from 5919 (80%) (termed responders), whereas 1450 (20%) could not be reached according to protocol (termed non-responders). See Figure 1.

The median age of the elderly participants was 65 years (range 60–82 years), of the young group was 45 years (range 15–59 years) and of all participants was 50 years (range 15–82 years).

For the participants attending the GSP and who were older than 60 years of age a total of 37% were continuous abstinent six month after the intended quit date. For the young, 35% were continuous abstinent after six months and for all the participants 36% were continuous abstinent after six month (*p* < 0.05). Among the elderly 38% of men and 36% of women were still abstinent after six months, whereas only 36% of the younger men and 33% of the younger women were abstinent after six months.

Participants among the elderly who had a smoking history of more than 20 pack-years (82%) had a continuous abstinence rate of 36% compared to those with <20 pack-years (18%) who had a continuous abstinence rate of 42% (*p* < 0.05). Among the young participants 55% had a smoking history of >20 pack-years and their abstinence rate was 33%.

A total of 5157 (70%) of the elderly were compliant with the GSP and their continuous abstinence rate after six months was 43% compared to an abstinence rate of only 19% for the 2837 participants that were not compliant with the program (*p* < 0.05). For the young, only 58% were compliant but the abstinence rate was similar (43%).

When the non-responders were included in the analyses—and categorized as having relapsed and therefore considered smokers—The continuous abstinence rates decreased in all three groups. Similarly, when non-responders were included the continuous abstinence rate after six months for the elderly was reduced to 30%, and for the young to 25% and for all to 26%.

The characteristics and abstinence rates of all three groups for responders only and for all are shown in Table 2.

The main differences between responders and non-responders in the group of the elderly were whether they were compliant with the program (responders 73%, non-responders 58%, *p* < 0.05), abstinent at end of program (responders 57%, non-responders 42%, *p* < 0.05) and whether they were in individual or group-based program (responders in group-based 85%, non-responders in group-based 82%, *p* < 0.05). Otherwise the two groups were equal and comparable in terms of gender, age, Fagerström and housing.

Table 3 shows the univariate and multivariate analysis with the variables for continuous abstinence after six months among the elderly. We found as significant factors in the final adjusted model: whether the participant lived with another adult (OR 1.10), if they had prior professional recommendation for smoking cessation (OR 1.12), if they were compliant with the program (OR 1.35) and whether they were abstinent at the end of the program (OR 13.3).

**Table 2:** Characteristics in numbers (*N*) and six months continuous abstinence rates given in percent (%) for all (including non-responders) and for responders only for both elderly, young and all.

Variables	Elderly: 60–82 Years.			Young: 15–59 Years.			All: 15–82 Years.		
	*N*	All *N* = 7369 (%)	Responders *N* = 5919 (%)	*N*	All *N* = 24,294 (%)	Responders *N* = 17,801 (%)	N	All *N* = 31,663 (%)	Responders *N* = 23,720 (%)
**Gender**									
*Male*	3138	31	38	9415	26	36	12,553	27	37
*Female*	4231	29	36	14,879	24	33	19,110	25	34
**Setting**									
*Hospital*	764	29	36	2321	25	35	3085	26	35
*Other*	6605	29	37	21,973	25	34	28,578	26	36
**Program**									
*Individual*	1116	31	41	3129	27	39	4245	28	39
*Group*	6178	29	36	20,833	24	33	27,011	25	34
**Smoking**									
*0-20 pack- years*	1,001	34	42	10,027	25	36	11,028	26	36
*>20 pack-years*	6,026	28	36	13,281	25	33	19,307	26	34
*Fagerström 1–4*	2951	32	39	7940	29	38	10,891	29	39
*Fagerström 5–10*	4193	27	34	15,580	22	31	19,773	23	32
*0-9 cig. per day.*	884	34	43	1942	33	44	2826	33	44
*10–19*	2,508	32	39	8650	26	36	11,158	28	37
*20–29*	2809	28	35	10,196	23	32	13,005	24	33
*30–39*	781	26	32	2356	21	29	3131	22	30
*+40*	387	24	31	1156	20	28	1543	21	29
Heavy smoker ^*^									
*Yes*	6157	28	36	16,893	24	32	23,050	25	39
*No*	1212	34	43	7401	28	38	8613	29	33
**Region**									
*Capital*	2698	28	35	9050	24	33	11,748	25	37
*Northern*	371	30	40	1145	26	36	1516	26	37
*Middle*	1610	29	37	5496	24	34	7106	25	35
*Southern*	1448	32	38	5212	26	35	6660	28	36
*Sealand*	1242	30	38	3391	25	34	4633	27	35
**Living with adult**									
*Yes*	3063	33	40	14,389	26	36	17,452	28	37
*No*	4238	27	34	9631	22	31	13,869	24	32
**Living with smoker**									
*Yes*	1932	30	36	8785	23	32	10,717	25	33
*No*	5382	29	37	15,322	26	35	20,704	27	36
**Professional recommend**									
*Yes*	2035	32	41	10,083	27	36	12,118	28	37
*No*	5334	28	35	14,211	24	32	19,545	25	33
**Compliant with program**									
*Yes*	5157	36	43	14,061	33	43	19,218	34	43
*No*	2212	14	19	10,233	13	19	12,445	13	19
**Employed**									
*Yes*	1267	31	40	17,335	27	36	18,602	27	36
*No*	5941	29	36	6321	20	29	12,262	24	33
**Attempts to quit**									
*No previous attempts*	3025	29	37	9310	24	33	12,335	25	34
*Previous attempts*	4124	30	37	14,591	26	35	18,715	27	35
**Abstinent at end of program**									
*Yes*	4120	48	57	11462	43	54	15582	44	55
*No*	2145	5	7	6380	4	7	8525	5	7

* Heavy smoker defined as: smoking ≥20 pack-year and/or consumption of ≥20 cigarettes a day and/or Fagerström nicotine dependence score of ≥7 points.

## 4. Discussion

This study showed a high continuous abstinence rate for participants over the age of 60 years in the Danish Gold Standard Program for smoking cessation. Participants over the age of 60 actually had significantly higher continuous abstinence rates both compared to the participant under the age of 60 years, but also to all participants in the GSP. This shows an age group motivated for quitting their smoking habits and sticking with it.

The significant variables for continuous tobacco abstinence can be grouped in modifiable or non-modifiable variables. Of the four variables that were significant in this study population, there was only one non-modifiable variable. This was whether the participant lived with another adult (OR 1.10), which can only be taken note of.

Two of the significant factors for a successful continuous abstinence were to do with the quality of the program and the motivation of the participants. Being compliant with the program, meaning attending the courses as planned, enhanced the chances for quitting the smoking habit (OR 1.35). This has also been shown for disadvantaged smokers, heavy smokers and pregnant smokers [18,19,20] and is of great importance for the individual smoking cessation sites. If the smoking cessation instructors can support the patient’s motivation and maintain good contact to the participants it is more likely they attend the planned meetings and thereby their success for quitting is greater. So the instructors giving the smoking cessation programs are of great importance. It could also be that it is the most motivated participant who are compliant with the program and thereby are better at quitting the tobacco.

Being abstinent at the end of the program is the greatest factor for continuous abstinence six months after (OR 13.3). This is not of great surprise, but it still gives a good inclination that if the smoking cessation programs are at their best, if the participants are motivated and therefore compliant with the program, more will be abstinent at the end of the program and therefore also continuous abstinent six months later. Together adding to the success of continuous abstinence. It is also interesting that of the participants who are still smokers at the end of the program, between 4%–7% report being abstinent after six months at follow-up (Table 2). This could be due to low motivation at end of program, smoking cessation date after end of program, or it could also possibly be reporting bias.

Also, professional recommendation did have an effect on continuous abstinence (OR 1.12). Even though the odds ratio is relatively small it still shows how important it is for general practitioners, hospital doctors and other health care personnel to inform patients about the risk of smoking, and ways to quit. Other studies, including a review of 42 trials, also show that life style consultations significantly increase the rate of smokers wanting to attend smoking cessation courses [21,22], and this in smokers who are not admitted to hospitals or are to be operated upon, so there could be good reason to believe that the “therapeutic opportunity” to encourage hospital admitted smokers are even greater than for the general population. It could be noted though that among fertile women (15–54 years) and among disadvantaged smokers attending the GSP the opposite has been shown with prior professional recommendation reducing the continuous abstinence rate. In these cohorts after controlling for other variables, such as pregnancy and other effect modifiers, the multivariate analysis showed a significant better continuous abstinence rate for the groups not receiving recommendation from health professionals prior to the smoking cessation program. This may be due to gender, educational and social related differences in attitudes and preferences of smoking cessation intervention, which has however not been investigated further [18,20].

**Table 3:** Primary outcome: Odds Ratios with 95% confidence intervals for continuous abstinence rates after six months for participants >60 years (non-responders seen as continuous smokers) for the univariate, multivariate and the final adjusted multivariate model.

Variables:		*N* = 7369	Univariate OR (CI)	Multivariate OR (CI)	Final adjusted OR (CI)
Gender:	Female	4231	1	1	--
	Male	3138	0.91 (0.82–1.01)	1.00 (0.88–1.35)	
Working:	No	5941	1	1	--
	Yes	1267	0.90 (0.79–1.03)	1.04 (0.88–1.23)	
Living with adult:	No	4238	1	1	1
	Yes	3063	1.25 (1.18–1.33)^*^	1.14 (1.00–1.29) ^*^	1.10 (1.00–1.23)^*^
Setting:	Hospital	764	1	1	--
	Other	6605	1.00 (0.85–1.15)	1.08 (0.87–1.34)	
Program:	Group based	6178	1	1	--
	Individual	1116	1.10 (1.05–1.26)^*^	1.03 (0.89–1.20)	
Heavy smoker^**^	No	1212	1	1	--
	Yes	6157	0.80 (0.71–0.91)^*^	0.92 (0.80–1.07)	
Professional recommendation:	No	5334	1	1	1
	Yes	2035	1.23 (1.10–1.37)^*^	1.11 (0.98–1.28)	1.12 (1.00–1.26)^*^
Compliant with program:	No	2212	1	1	1
	Yes	5157	3.56 (3.12–4.07)^*^	1.34 (1.10–1.65) ^*^	1.35 (1.03–1.62)^*^
Non-smoker at end of program:	No	2037	1	1	1
	Yes	3951	17.1 (13.9–21.1)^*^	13.8 (10.5–16.8) ^*^	13.3 (10.7–16.4) ^*^

OR Odds Ratio, CI Confidence Interval, ^*^ significant with *P* < 0.05. ^**^ Heavy smoker defined as: smoking ≥20 pack-year and/or consumption of ≥20 cigarettes a day and/or Fagerström nicotine dependence score of ≥7 points.

Whether the participants attended group or individual programs was not a significant variable in the multivariate analysis for the elderly, even though other studies from the Danish Smoking Cessation Database show that the program had a significant influence in increasing the numbers of continuous abstinence, favoring individual programs over group programs [18,19,20], the opposite has also been shown [23].

Being older than 60 years should never imply that smoking cessation is too late. Back in the 90’s several predictors was identified in the elderly population highlighting the variables increasing smoking cessation in this specific population group. It was found that hospitalized patients were more inclined to smoking cessation, as well as being married to non-smoking spouse, motivation was important and their prior attempts of quitting was significant variables [24]. Designing programs targeted for the elderly population does seem to have potential for success, because when attending smoking cessation intervention programs designed to all, it is seen that smokers over the age of 60 years who participate, succeed better than younger participants. Cessation rates after four weeks are significantly higher for the elderly than the younger participants [25] and this also applies after one year [23]. So one might assume that if smoking cessation programs for the elderly were even more targeted, the quit rates could be even higher.

Using the knowledge obtained from registry-based studies can heighten the quality of the smoking cessation programs. Smoking cessation has plenty of health benefits for both the elderly as well as the young. Cardiovascular and even non-tobacco related cancer-mortality is diminished in former smokers compared to current smokers and some studies even show that the cardiovascular mortality in former smokers is similar to that of never-smokers [26,27].

We have to consider that there are both limitations as well as strengths to this study. The limitations of this study are in the selection of data, the registration of the data and possible selection bias. Being a registry-based study it has limitations compared to a randomized trial. Also, the abstinence rates are all based on self-reported data. This could give an overestimation of abstinence rates of 3%–6% [21,23]. The strengths of this study lie especially in the high number of participants included. Also, approximately 90% of all smoking cessation intervention sites in Denmark report to the Danish Smoking Cessation Database, giving a realistic characteristic of the participants with the normal background population and thereby minimizing the selection bias. Since the instructors are educated according to the same program this gives consistency in the meetings and conversations with the participants all over the country adding strength to the data collected. Statistical strength is also added since all non-responders are considered still-smokers when included in the analyses.

## 5. Conclusions

Participants following the GSP for smoking cessation and who were over the age of 60 years old had a continuous abstinence rate after six months of 37%. This was a higher abstinence rate than participants less than the age of 60 and also higher than for all participants registered in the Danish Smoking Cessation Database. The significant variables for continuous smoking cessation six months after the program were living with another adult, having prior professional recommendation on smoking cessation and the risks of continuous smoking, being compliant with the program and being abstinent at the end of the program.

It is never too late for health professionals to recommend, educate and influence patients for attending smoking cessation programs, even if they are over 60 years of age. This actually only increase the success rate for quitting compared to the younger age groups and they still gain important health benefits compared to continuous smokers.
